# Unraveling the role of microRNAs: potential biomarkers for gestational diabetes mellitus revealed through RNA sequencing analysis

**DOI:** 10.1007/s00404-024-07518-x

**Published:** 2024-05-30

**Authors:** Huizhen Lin, Xiao Chen, Lisui Wang, Tang Zhu, Xiaohui Feng, Xiaomei Liu, Haiying Chen, Si Pan

**Affiliations:** 1 Department of Clinical Laboratory, The First Hospital of Putian, Teaching Hospital, Fujian Medical University, Putian, 351100 China; 2 Key Laboratory of Translational Tumor Medicine in Fujian Province, Putian University, School of Basic Medicine Science, Putian, 351100 Fujian China

**Keywords:** Gestational diabetes mellitus, MicroRNAs, Biomarkers, Peripheral blood, RNA sequencing

## Abstract

**Background:**

Gestational diabetes mellitus (GDM) poses significant health risks for both mothers and children, contributing to long-term complications such as type 2 diabetes and cardiovascular disease. This study explores the potential of microRNAs (miRNAs) as biomarkers for GDM by analyzing peripheral blood samples from GDM patients.

**Method:**

Ten samples, including peripheral blood from 5 GDM patients and 5 controls, were collected to perform the RNA sequencing analysis. Differentially expressed miRNAs were further validated by quantitative real-time polymerase chain reaction.

**Results:**

A total of 2287 miRNAs were identified, 229 of which showed differential expression. Validation by qRT-PCR confirmed significant up-regulation of miR-5193, miR-5003-3p, miR-3127-5p, novel-miR-96, miR-6734-5p, and miR-122-5p, while miR-10395-3p was down-regulated. Bioinformatics analyses revealed the involvement of these miRNAs in pathways associated with herpes simplex virus 1 infection.

**Conclusion:**

This study provides insights into the differential expression of miRNAs in GDM patients and their potential roles in disease pathogenesis. It suggests that the differentially expressed miRNAs could serve as potential biomarkers for GDM, shedding light on the complex molecular mechanisms involved.

**Supplementary Information:**

The online version contains supplementary material available at 10.1007/s00404-024-07518-x.

## What does this study add to the clinical work

**Table:** 

This study identifies specific miRNAs that are differentially expressed in gestational diabetes mellitus (GDM) patients, suggesting their potential as biomarkers for GDM diagnosis and highlighting their roles in the disease pathogenesis, particularly in pathways associated with herpes simplex virus 1 infection.	

## Introduction

Gestational diabetes mellitus (GDM) is a common complication of pregnancy, referring to diabetes diagnosed for the first time in the mid or late stages of pregnancy, which is neither pre-existing type 1 nor type 2 diabetes [1, 2]. Risk factors for GDM include overweight/obesity, Westernized diet and micronutrient deficiencies, advanced maternal age, insulin resistance, and/or family history of diabetes [3–5]. Although most GDM patients can return to normal after delivery, poor glycemic control in some cases can lead to serious risks and long-term damage [6]. These damages include the development of type 2 diabetes (T2DM) and cardiovascular disease (CVD), the increased risk of preeclampsia and the increased need for cesarean section in mothers, as well as the complications in newborns, such as preterm birth, polyhydramnios, macrosomia, shoulder dystocia, admission to the neonatal intensive care unit, neonatal respiratory distress syndrome, neonatal hypoglycemia, and hyperbilirubinemia. In addition, there is an increased risk of future obesity, CVD, T2DM, and/or GDM in children [1, 3, 4, 7]. With the increasing trend toward obesity, the incidence of GDM is also increasing significantly. According to 2017 data from the International Diabetes Federation, approximately 14% of pregnant women worldwide (about 18 million people) are affected by GDM each year [8]. The prevalence of GDM is even higher in Southeast Asia, reaching 24.2%. Although 70–85% of GDM patients can be treated through appropriate physical activity, diet, and lifestyle changes, 15–30% of patients still require medication [9]. These medications include insulin and oral glucose-lowering drugs [10]. Although newer oral antidiabetic drugs such as glipizide and metformin have emerged, there are still concerns about their long-term safety for both mother and child [3, 4]. Currently, there is no effective cure or prevention strategy for GDM. One reason is that the molecular mechanisms of GDM are still unclear [1].

Non-coding RNAs (ncRNAs) account for about 60% of the transcriptional products in the human genome and play a central regulatory role in many physiological and pathological processes, including cell proliferation, differentiation, apoptosis, and disease occurrence and development [11]. ncRNAs are divided into two major classes: small ncRNAs, such as microRNAs, which are less than 200 nucleotides in length, and long ncRNAs (lncRNAs), which are longer than 200 nucleotides [12, 13]. MiRNAs play a leading role in RNA silencing. MiRNA function by pairing with target mRNA sequences, which can be located in coding regions or intronic regions of non-coding transcripts, and even in exonic regions [14]. According to the latest statistics from 2018, 2654 mature microRNA sequences have been discovered in humans [15]. The functions of many miRNAs are still being defined. Existing research data has shown that a large number of miRNAs play a key role in gene regulation. Their dysregulation is associated with various diseases, from cancer to metabolic diseases [16, 17]. Multiple studies have shown that obesity, T2DM, and cardiovascular disease are associated with miRNA dysregulation or dysfunction, potentially playing a role in regulating beta-cell function and quality, as well as metabolic processes [18, 19]. According to the whole genome analysis, there are more than 600 miRNAs present in the placenta, which may play an important role in pregnancy and GDM [18, 20, 21]. Cao et al. found that the expression of miR-98 in placental tissue of pregnant women with GDM at 37–40 weeks was significantly higher than that of normal pregnant women (*n* = 202). MiR-98 can directly regulate the transcription factor methyl-CpG binding protein 2, interfere with glucose uptake in GDM by controlling the activity of transient receptor potential cation channel subfamily C member 3 [22]. Nair et al. found that compared to normal controls, there were 13 up-regulated miRNAs and 14 down-regulated miRNAs in GDM. It should be noted that the predicted target genes of these miRNAs are largely involved in glucose metabolism, including the phosphatidylinositol 3-kinase (PI3K)/protein kinase B (AKT) signaling pathway [23, 24]. Li et al. showed that miR-96 was down-regulated in GDM placental tissue and may alter beta-cell function by targeting p21 (RAC1) activated kinase 1 [25, 26].

Given that a single miRNA can target hundreds of mRNAs, and a specific target mRNA is typically controlled by several different miRNAs, to study such complex network control mechanism of miRNAs in GDM is a time-consuming and inefficient choice using traditional biomedical research methods. This study used RNA sequencing transcriptomics analysis, which has the ability to study large amounts of data using computer technology, to compare and analyze the variations of plasma miRNAs in GDM patients and normal pregnant women. It provides scientific data and evidence for further research and explanation of the complex pathological mechanisms of GDM. These abnormally expressed miRNAs in GDM may also become early diagnostic biomarkers or therapeutic targets for GDM in the future.

## Methods

### Ethics and subjects

The GDM patients were all recruited from the outpatient clinic of The Frist Hospital of PuTian City. Normal controls exhibited normal glucose tolerance. GDM diagnosis was based on the 75 g oral glucose tolerance test (OGTT), with the diagnosis criteria fasting plasma glucose ≥5.1 mmol/L, 1 h plasma glucose ≥10.0 mmol/L or 2 h plasma glucose ≥8.5 mmol/L following the 75 g OGTT. The patients were excluded with the following criteria: known pre-existing diabetes mellitus before pregnancy; history of chronic renal disease or hepatic dysfunction; any significant medical condition that could interfere with glucose metabolism; multiple pregnancies (e.g., twins or triplets); incomplete or missing data from the oral glucose tolerance test (OGTT). The study protocol received approval from the institutional ethics committee, and informed consent was obtained from all participants. The confidentiality and privacy of participant information were strictly maintained throughout the study.

### Peripheral blood collection for miRNA sequencing

Ten samples were collected, including peripheral blood from five GDM patients and five controls. Blood was stored in an anticoagulant tube containing EDTA, immediately frozen in liquid nitrogen, and stored at −80 ℃ for subsequent tests.

### RNA extraction and miRNA sequencing

Total RNA was extracted from peripheral blood using the miRNeasy Mini Kit (Cat No. 217004, Qiagen, CA, USA). Total RNA quantity and purity were analyzed using RNA Nano 6000 Assay Kit of the Agilent Bioanalyzer 2100 System (Agilent Technologies, CA, USA) with RIN number ≥ 8. Then, approximately 1.5 μg of total RNA was used to build a small RNA library using the Illumina VAHTSTM Small RNA Library Prep Kit (Illumina Inc., CA, USA). Subsequently, unique sequences with length of 18–30 nt were mapped to specific species precursors with miRbase v22 by BLAST search. All procedures were performed by Biomarker Technologies.

### qRT-PCR for further verification

Total RNA was extracted from peripheral blood using the miRNeasy Mini Kit (Cat No. 217004, Qiagen, CA, USA) and U6 was used as an internal reference to normalize miRNA expression. Total RNA from each sample was reverse-transcribed using M-MuLV Reverse Transcriptase (Cat No. P7040L, Enzymatics, USA), according to the manufacture’s protocol. qRT-PCR was performed with a 2× PCR master mix (Cat No. AS-MR-006-5, Arraystar, MD, USA) to quantify miRNA expression. After pre-denaturation at 95 ℃ for 10 min, 40 PCR cycles were performed (95 ℃ for 10 s, and 60 ℃ for 60 s). The expression of target genes was analyzed using the 2^−ΔΔCt^ method [27]. The primer information for U6 and miRNAs is shown in Table S1.

### Statistical analyses

Data were analyzed using SPSS 22.0 software (IBM Corporation, Armonk, NY, USA) and GraphPad Prism version 8.0 (GraphPad Software Inc., San Diego, CA, USA). Variables were expressed as the mean ± standard deviation. Differences between groups were analyzed using Student’s *t* test. *P* < 0.05 was considered statistically significant.

### Bioinformatics analyses

The differential expression analysis of two groups was performed using the DESeq2 R package (1.10.1). miRNAs with |log2(FC)| ≥ 0.58, and *P* < 0.05 were assigned as differentially expressed. The target genes of the differentially expressed miRNAs were predicted with selectively predicted algorithms in two independent online software programs: miRanda v3.3a and TargetScanS v 5.0 software. Novel miRNA prediction was carried out using miRDeep2 [28]. Gene Ontology (GO) enrichment analysis of the differentially expressed genes was implemented by the ClusterProfiler R packages based on Wallenius non-central hyper-geometric distribution. Kyoto Encyclopedia of Gene and Genomes (KEGG) pathway analysis was carried out to investigate the potential significant pathways (http://www.genome.jp/kegg/) through the KOBAS software.

## Results

### Clinical characteristics

A total of ten women were included in the study, five with normal healthy pregnancies and five with GDM. Maternal demographics are detailed in Table 1. Although no change in HbA1c between groups was observed in the first trimester of pregnancy ruling out pre-existing diabetes mellitus, the 1 h OGTT test revealed higher blood glucose concentrations in GDM versus normal pregnancies (*p* = 0.003). There were no significant differences in BMI, age, weight, or height between the two groups.

**Table 1:** Basic data of CTLs and GDMs

	CTLs	GDMs	*t *Value	*P *Value
Number	5	5		
Age (years)	26.60 ± 3.647	29.60 ± 2.074	1.599	0.148
BMI	23.000 ± 3.374	21.800 ± 2.035	0.681	0.515
Weight (kg)	56.180 ± 5.627	59.520 ± 9.308	0.687	0.512
Height (cm)	155.600 ± 5.177	162.200 ± 4.604	2.13	0.066
GTT (0 h) (mmol/L)	4.524 ± 0.255	4.626 ± 0.481	0.419	0.687
GTT (1 h) (mmol/L)	6.934 ± 1.316	10.162 ± 1.036	4.309	0.003
GTT (2 h) (mmol/L)	6.380 ± 1.239	8.210 ± 1.967	2.766	0.049

Note: *BMI* body mass index, *GTT* glucose tolerance test, *CTLs* normal controls, *GDMs* gestational diabetes mellitus

### Identification of differentially expressed miRNAs

**Fig. 1:**
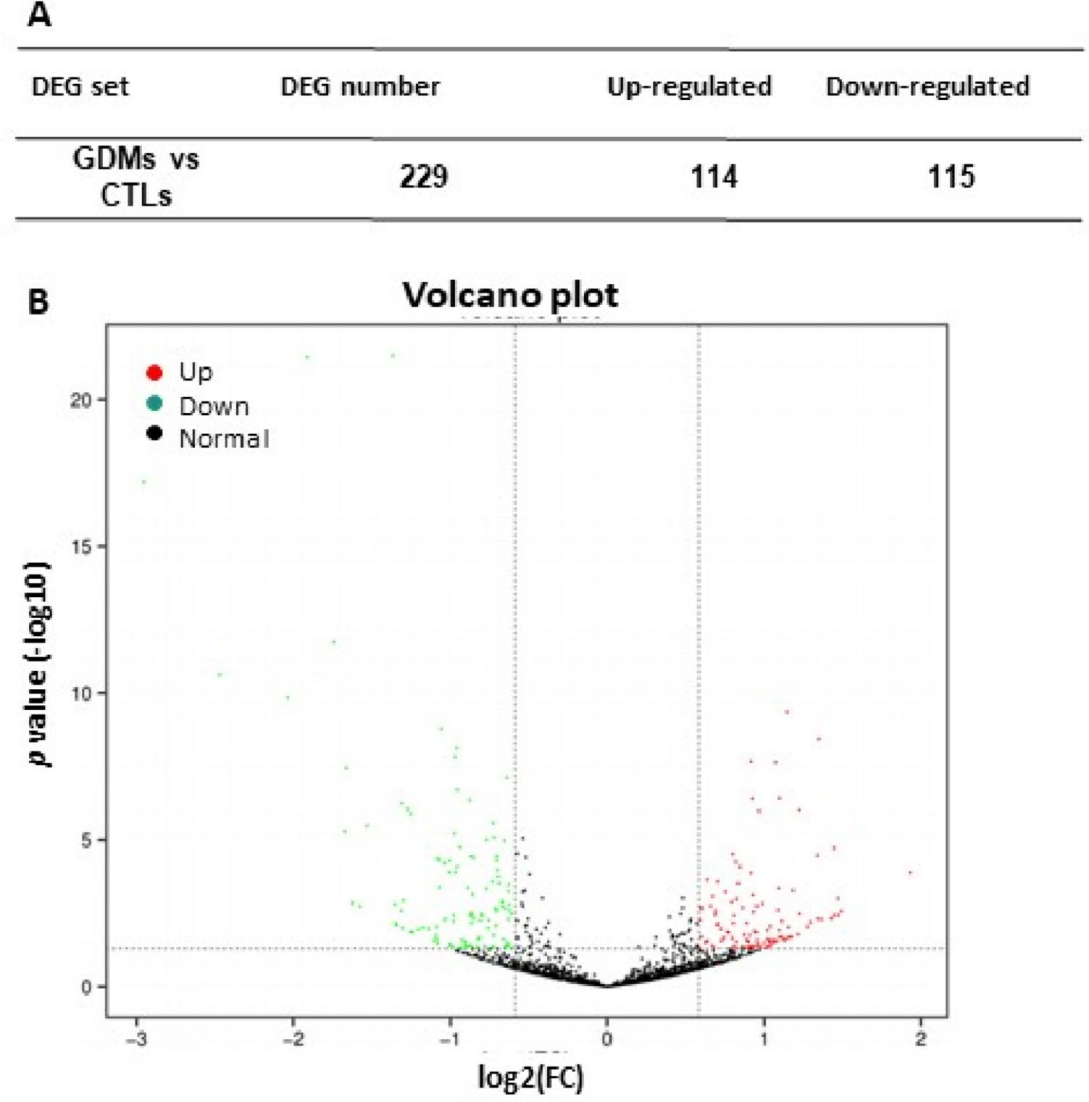
Volcano plot of differentially expressed miRNA. **A** Statistics on DE-miRNA in each set. **B** Volcano plots showing significantly different expression of miRNA between the groups. Red points, up-regulated miRNA; green points, down-regulated miRNA. *FC* fold changes

A total of 2287 miRNAs were identified, including 1420 known miRNAs in miRbase and 867 novel miRNAs predicted by miRDeep2. In total, 229 differentially expressed miRNAs were determined to have a |log2(FC)| ≥  0.58, and *P* < 0.05 by DESeq2 R package (1.10.1); 114 miRNAs were up-regulated and 115 miRNAs were down-regulated (Table S2). Volcano plot (Fig. 1) represented the significant differences in miRNA expression between the control (CTL) and GDM groups.

### Validation of differentially expressed miRNA by qRT-PCR

**Fig. 2:**
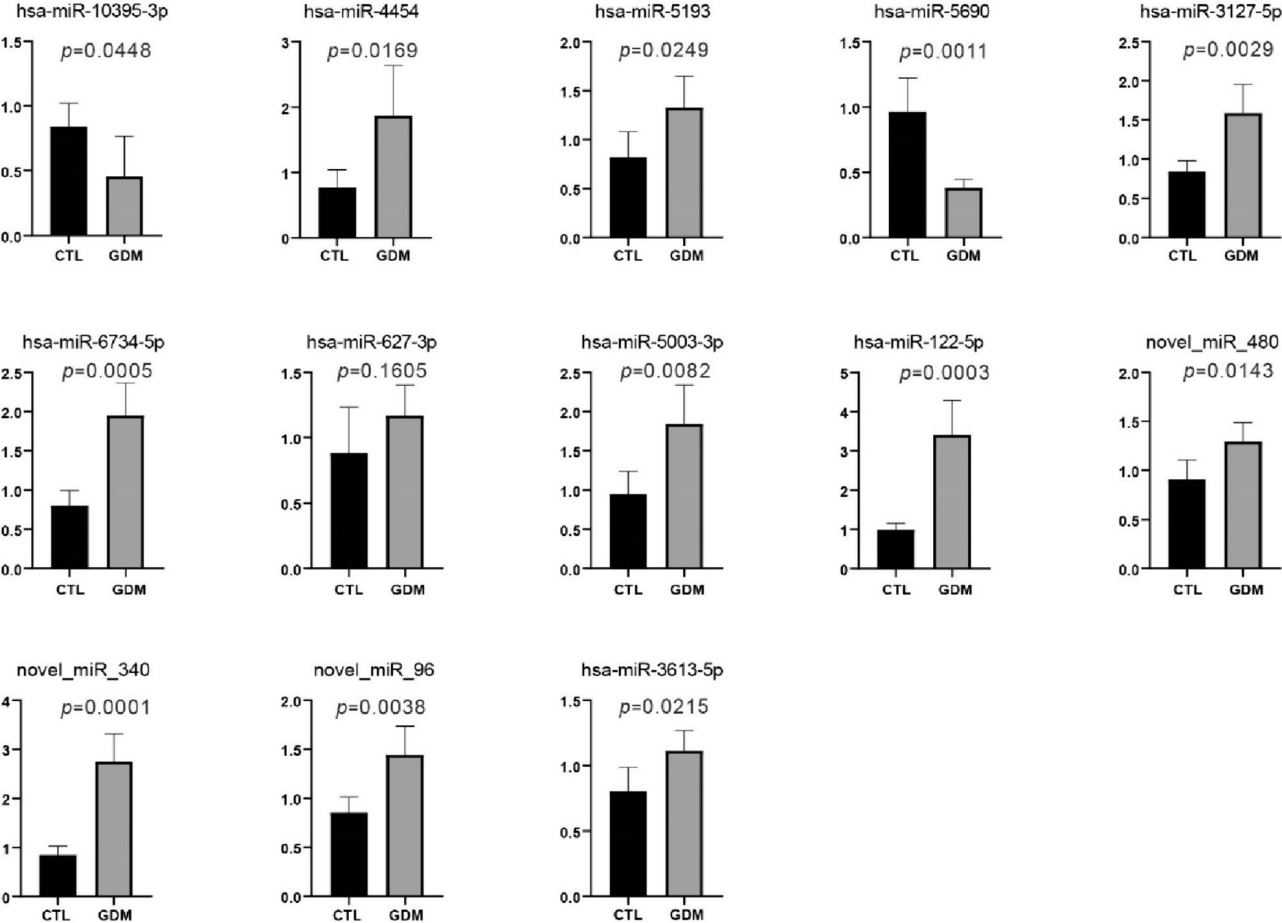
Validation of the differential expression of key miRNAs by qRT-PCR. The Y-axis shows the relative expression level. The X-axis shows different sets. *GDM* gestational diabetes mellitus, *CTL* control

Among the differentially expressed miRNAs mentioned above, 13 significant differentially expressed miRNAs (7 up-regulated miRNAs and 6 down-regulated miRNAs) with |log2(FC)| > 1.4 were selected for validation by qRT-PCR (Table 2). As shown in Fig. 2, the expression levels of miR-5193, miR-5003-3p, miR-3127-5p, novel-miR-96, miR-6734-5p, and miR-122-5p in peripheral blood of GDM patient were significantly up-regulated (*P* < 0.05), and the expression levels of miR-10395-3p were significantly down-regulated (*P* < 0.05). However, the qRT-PCR results of other selected miRNAs were not consistent with the next-generation sequencing results.

**Table 2:** Top different miRNAs between GDMs and CTLs

miRNA	*P* value	FDR	log2(FC)	Regulated
hsa-miR-10395-3p	6.22E−18	4.74E–15	–2.953160943	Down
hsa-miR-4454	2.41E–11	1.10E–08	–2.468947244	Down
novel_miR_480	1.40E–10	5.33E–08	–2.035840923	Down
hsa-miR-3613-5p	3.52E–22	4.03E–19	–1.91113531	Down
novel_miR_340	1.76E–12	1.01E–09	–1.743279814	Down
hsa-miR-627-3p	3.25E–06	0.000285594	–1.52948267	Down
hsa-miR-5193	0.000128843	0.005456747	1.929473327	Up
hsa-miR-5003-3p	0.002595505	0.056532572	1.491680168	Up
hsa-miR-3127-5p	0.003701499	0.071395845	1.472351339	Up
hsa-miR-5690	0.000980376	0.02874512	1.470261862	Up
novel_miR_96	0.003655407	0.071395845	1.445823692	Up
hsa-miR-6734-5p	1.89E–05	0.001272093	1.444353213	Up
hsa-miR-122-5p	0.004604296	0.086311677	1.427898387	Up

*P* < 0.01; |log2(FC)| > 1.4

### The function of the target genes of miRNAs

**Fig. 3:**
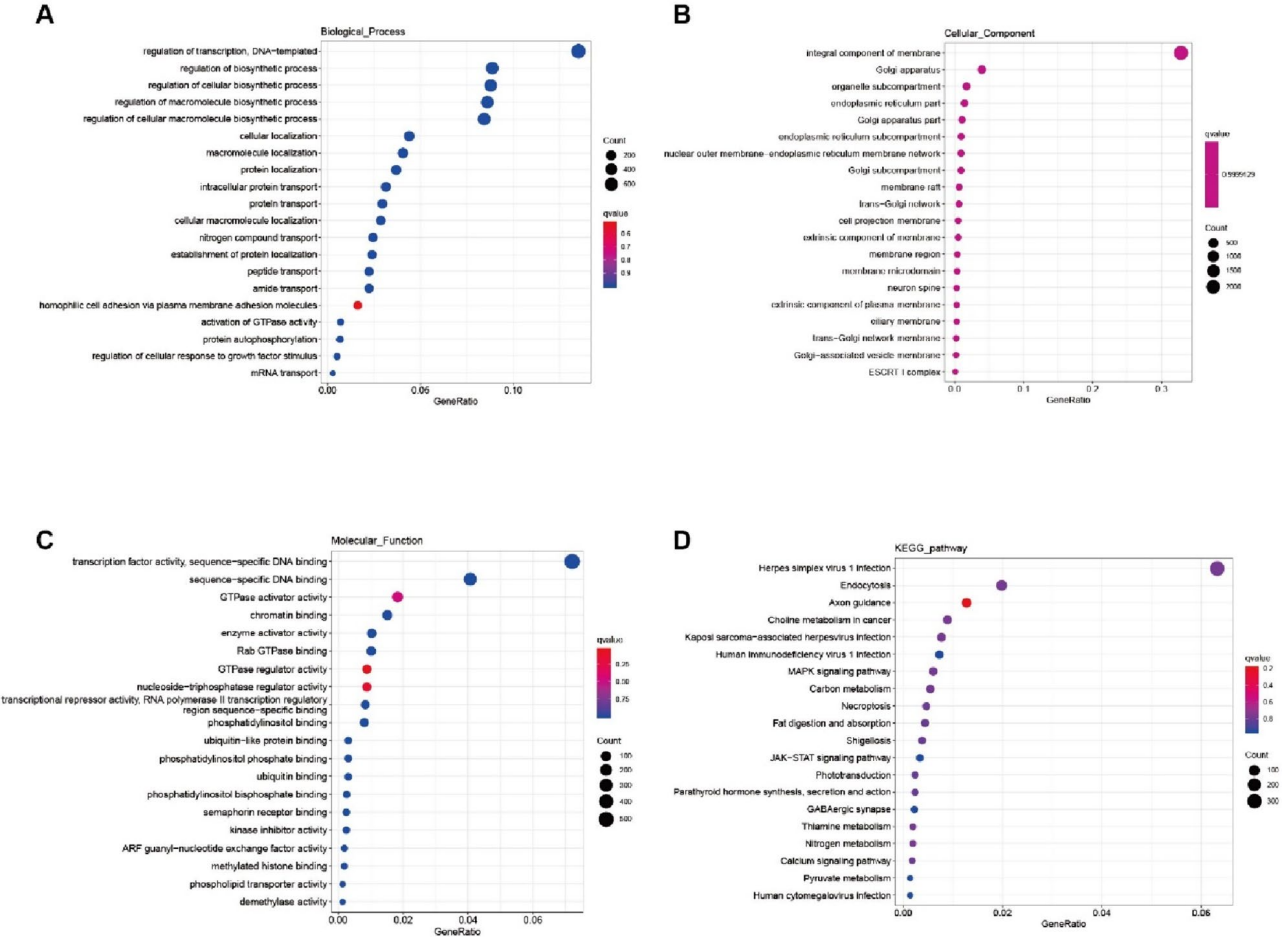
Function analysis of target genes of miRNAs. **A, B** and **C** The most enriched GO terms of target genes of miRNA. X-axis represents the gene ratio, and Y-axis represents the GO terms. **D** Enrichment analysis of target gene KEGG pathway. X-axis represents the enriched factor and Y-axis represents the KEGG pathways. The color of the dot indicates the different *q*-values. The size of the dot indicates the gene number

Target genes of differentially expressed miRNAs were predicted by the miRanda and TargetScanS software. A total of 11,046 target genes were predicted. Gene Ontology (GO) and Kyoto Encyclopedia of Gene and Genomes (KEGG) pathway analyses were conducted to explore the main functions of target genes (Fig. 3). GO analysis of target genes showed that the most potential target genes are involved in the regulation of transcription, biosynthetic process, integral component membrane, and DNA binding (Fig. 3A–C). Regulation of transcription and biosynthetic process were in biological processes, integral component membrane was encompassed in cellular component, and DNA binding was involved in molecular function. KEGG pathways for these genes were mainly enriched in the herpes simplex virus 1 infection (Fig. 3D).

## Discussion

GDM is the most common pregnancy complication that imposes a serious short- and long-term health risk to mother and child. While most GDM patients can recover afterward, poor glycemic control in some cases may result in the development of type 2 diabetes and cardiovascular diseases [6]. An effective therapy that can reduce the incidence of GDM is a major research priority for public health. Many molecular biomarkers for GDM have been investigated, including single-nucleotide polymorphisms (SNPs), metabolites, miRNAs, and proteins.

With the development of molecular biotechnology, non-coding RNA have received increasing attention in recent years. miRNAs are a subset of non-coding RNAs that act as negative regulators in gene expression. Many studies have shown that miRNA is widely involved in cell growth and development, metabolism, and apoptosis, and is strongly associated with human diseases. Over the years, evidence has increasingly shown that miRNA dysregulation has been linked to diabetes. It was reported that miRNA play an important role in type 1 and type 2 diabetes, including in beta-cell biology, insulin resistance, and diabetes complications [29]. In recent years, several miRNAs up-regulated in GDM patients have been identified. miR-29a, its serum expression was significantly down-regulated in pregnant women with GDM [30]. miR-657 affects macrophage-mediated immunity and inflammation in GDM [31]. miR-770-5p and miR-96 play protective roles in GDM by contributing to β-cell proliferation [26, 32]. Serum aberrant expression of miR-132 may exert a protective role against GDM by reducing the inhibition of high glucose in trophoblast cell proliferation [33]. These findings clearly establish the importance of serum miRNA in GDM patients. Moreover, miRNA can be collected from peripheral blood. Therefore, detecting the expression of miRNA in peripheral blood is a novel and simple method for GDM.

In this study, we collect peripheral blood of GDM patients to identify potential miRNA biomarker. A total of 2287 miRNAs were identified, including 229 differentially expressed genes. We performed qRT-PCR on 13 differentially expressed miRNAs based on our predicted sequences. The results showed that the expression of partial selected miRNAs was in line with the expression in sequencing. The expression levels of miR-5193, miR-5003-3p, miR-3127-5p, novel-miR-96, miR-6734-5p, and miR-122-5p in peripheral blood of GDM patients were significantly increased (*P* < 0.05), and the expression levels of miR-10395-3p were significantly decreased (*P* < 0.05). It has been shown that miR-5193 plays a significant role in prostate cancer and ovarian cancer [34, 35]. EIF4A3-regulated circ_0087429 can reverse EMT and inhibit cervical cancer progression via miR-5003-3p-dependent up-regulation of OGN expression [36]. The involvement of miR-3127-5p in preeclampsia has been documented, attributed to the fact that overexpression of its target gene HOXA7 can reverse the effects of miR-3127-5p in trophoblast cells [37]. The aberrant functionality of trophoblast cells is a crucial factor in the occurrence of preeclampsia. A previous study suggested that miR-6734-5p could be a notable miRNA associated with high-grade serous ovarian cancer [38]. miR-10395-3p was poorly studied, and currently, its dysregulation has only been supposed to play a significant role in lung cancer [39]. Unfortunately, in even GDM diabetes models, these miRNAs have not been reported to have relevant effects. This study firstly shows that these miRNAs may have essential roles in GDM. miRNA showing dysregulated expression in GDM might represent potential therapeutic targets for novel interventions. Further research is needed to validate the diagnostic, prognostic, and therapeutic utility of the identified miRNA biomarkers in larger patient cohorts and diverse populations.

Previously, miR-122-5p showed 2.55-fold increase in the T2D-liver cancer [40]. miRNA profiles in extracellular vesicles from GDM showed that miR-122-5p showed significantly higher levels in GDM cases than in controls [41]. In the insulin receptor signaling pathway, increased expression of miR-122-5p is predicted to inhibit insulin binding to the insulin receptor protein [41]. In this study, miR-122-5p was significantly up-regulated in GDM cases (*P* < 0.05), which is consistent with previous report. It indicates that they may play an important role in the pathogenesis of GDM and are expected to become new molecular biomarkers of GDM.

In recent years, extensive studies have shown that several key pathways involved in the development of GDM are associated with the pathophysiology of T2DM. The nuclear factor-kappa light chain enhancer of activated B cells (NF-κB) signaling pathway has critical role in gene expression for immune and inflammatory responses [42]. The importance of the NF-κB pathway extends to GDM and has been reported in the GDM placenta with increased NF-κB mRNA [43]. It is also worth noting that increased levels of chorionic gonadotrophin (CG) in GDM can impair insulin signaling in adipocytes through the NF-κB pathway [44]. Toll-like receptors (TLRs) are key surface molecules with and an essential role in triggering and inflammatory innate immune response [45]. Tangeras et al. showed that most TLRs are functionally active in human placenta. GDM is associated with increased expression of MyD88 and TLR4 mRNA in the placenta [43, 46]. The PI3K/mTOR pathway is required for survival in environments with variable nutrient availability. Compared with the expression of mTOR pathway in placentas of normal term babies and GDM babies, the increased expression of the ribosomal protein p-p70S6K, a downstream component of the mTOR signaling network, indicates that mTOR plays a role in the observed pathology in the placenta of GDM births [47]. Glycogen synthase kinase 3 (GSK3), a serine/threonine protein kinase, was originally found to have a role in the storage of glucose in glycogen [48]. Women with GDM showed significantly reduced GSK3β serine phosphorylation in their skeletal muscle and omental adipose tissue [49]. Similarly, AMPK activity was also significantly reduced in skeletal muscle and adipose tissues of GDM patients [50, 51]. In pregnant adipose tissue, the inflammasome processes IL-1β secretion in TLRs and pro-inflammatory cytokine signaling pathways [52]. Recently, it also reported that placental VEGF and CD31 expression in pregnancies complicated by GDM show influence on pregestational BMI and gestational weight gain in women with GDM [53].

We performed GO and KEGG pathway analyses to explore the biological functions and potential pathways in genes of differentially expressed miRNAs. In the GO enrichment analysis, the most significant biological processes are regulation of the transcription and biosynthetic process, the predominant cellular component is the integral membrane component, and the principal  molecular functions are transcription factor activity and sequences-specific DNA binding. KEGG pathway analysis showed that the most significantly enriched pathway is the herpes simplex virus 1 infection pathway. Type 1 herpes simplex virus (HSV-1) is one of the most common human antigens, infecting billions of people. Each year, between 250,000 and 500,000 of every million virus-infected individuals experience severe symptoms, leading to herpes simplex encephalitis, and most of these unfortunate cases occur in children under the age of 3 years [54]. Through RIG-I affinity purification and RNA sequencing of cells infected with the herpes simplex virus, it was revealed that small non-coding RNAs, especially RNA5SP141, constitute a class of intracellular ligands for RIG-I [55]. Gene mutations in nucleic acid sensing components such as TLR3 or its downstream signaling molecules (including UNC93B1, TLR3, TRIF, TRAF3, TBK1, IRF3, etc.) are one of the causes of herpes simplex encephalitis. It was reported that TLR3 ligand significantly increased the expression of a number of inflammatory markers in the skeletal muscle of pregnant woman [56]. Additionally, treatment of skeletal muscle of pregnant women with poly(I:C) could result in a decrease in glucose uptake [57]. All these findings strongly indicate that differentially expressed miRNAs in GDMs, along with their target genes, play a crucial role in the pathogenesis of the disease.

## Conclusion

In conclusion, this study provides insights into the differential expression of miRNAs in GDM patients and their potential roles in disease pathogenesis. Identified miRNAs, especially miR-5193, miR-5003-3p, miR-3127-5p, novel-miR-96, miR-6734-5p, miR-122-5p, and miR-10395-3p, may serve as novel biomarkers for GDM. More research is warranted to elucidate the specific functions and mechanisms of these miRNAs in GDM, paving the way for improved diagnosis and therapeutic interventions.

### Supplementary Information

Below is the link to the electronic supplementary material.

Supplementary file1 (DOCX 34 KB)

## Data Availability

The data sets used and analyzed during this study are available and under the domain of the corresponding author (plar000@163.com).
