# Supplementary material for: Unraveling the role of microRNAs: potential biomarkers for gestational diabetes mellitus revealed through RNA sequencing analysis
Source: Arch Gynecol Obstet. 2024 May 30;310(2):1255–64. doi: 10.1007/s00404-024-07518-x (PMC11258170; doi:10.1007/s00404-024-07518-x)
Supplement: Supplementary file 1 — Supplementary file1 (DOCX 34 KB) [file 404_2024_7518_MOESM1_ESM.docx]

**Supplemental materials**

**Table S1 The primer information for U6 and the miRNAs.**

| **Name** | **primer** |
| --- | --- |
| U6 | F:5’GCTTCGGCAGCACATATACTAAAAT3’  R:5’CGCTTCACGAATTTGCGTGTCAT3’ |
| hsa-miR-10395-3p | GSP:5’GGGGGGATGTATTCGTACTGT3'  R:5’GTGCGTGTCGTGGAGTCG3’ |
| hsa-miR-4454 | GSP:5’GGCACGATCCGAGTCACG3’  R:5’GTGCGTGTCGTGGAGTCG3’ |
| hsa-miR-5690 | GSP:5'TCCCCCTCAGCTACTACCTCT3’  R:5'GTGCGTGTCGTGGAGTCG3’ |
| hsa-miR-122-5p | GSP:5’GGGTGGAGTGTGACAATG3'3'  R:5'CAGTGCGTGTCGTGGAGT3' |
| hsa-miR-3127-5p | GSP:5'GGAATCAGGGCTTGTGGAAT3'  R:5’GTGCGTGTCGTGGAGTCG3’ |
| novel_miR_96 | GSP:5'GAAAAGACCCGCTTCCTCC3'  R:5’GTGCGTGTCGTGGAGTCG3’ |
| novel_miR_340 | GSP:5'GGGGGAGTGCCTGTAGTC3'  R:5’GTGCGTGTCGTGGAGTCG3’ |
| hsa-miR-3613-5p | GSP:5'GGGGGGGTGTTGTACTTTTT3'  R:5’GTGCGTGTCGTGGAGTCG3’ |
| hsa-miR-5003-3p | GSP:5'GGGGGGTACTTTTCTAGGTTG3'  R:5’GTGCGTGTCGTGGAGTCG3’ |
| hsa-miR-627-3p | GSP:5'GGGGGGTCTTTTCTTTGAG3'  R:5’GTGCGTGTCGTGGAGTCG3’ |
| hsa-miR-5193 | GSP:5'GGGGTCCTCCTCTACCTCAT3'  R:5’GTGCGTGTCGTGGAGTCG3’ |
| novel_miR_480 | GSP:5'GGGTTCTGAGGCTGGAGG3'  R:5’GTGCGTGTCGTGGAGTCG3’ |
| hsa-miR-6734-5p | GSP:5'GGTTGAGGGGAGAATGAGG3'  R:5’GTGCGTGTCGTGGAGTCG3’ |

**Table S2 Differentially expressed miRNA between GDMs and CTLs**

| **miRNA** | **Pvalue** | **FDR** | **log_2_FC** | **regulated** |
| --- | --- | --- | --- | --- |
| hsa-miR-10395-3p | 6.22E-18 | 4.74E-15 | -2.95316 | down |
| hsa-miR-4454 | 2.41E-11 | 1.10E-08 | -2.46895 | down |
| novel_miR_480 | 1.40E-10 | 5.33E-08 | -2.03584 | down |
| hsa-miR-3613-5p | 3.52E-22 | 4.03E-19 | -1.91114 | down |
| novel_miR_340 | 1.76E-12 | 1.01E-09 | -1.74328 | down |
| hsa-miR-1246 | 5.07E-06 | 0.00043 | -1.67136 | down |
| novel_miR_311 | 3.57E-08 | 5.82E-06 | -1.66232 | down |
| novel_miR_249 | 0.001421 | 0.038309 | -1.62194 | down |
| novel_miR_830 | 0.00184 | 0.045437 | -1.57759 | down |
| hsa-miR-627-3p | 3.25E-06 | 0.000286 | -1.52948 | down |
| hsa-miR-27a-3p | 3.05E-22 | 4.03E-19 | -1.3675 | down |
| novel_miR_589 | 0.001537 | 0.040403 | -1.35318 | down |
| hsa-miR-449a | 0.006932 | 0.111641 | -1.34641 | down |
| novel_miR_29 | 0.007909 | 0.123046 | -1.34352 | down |
| novel_miR_391 | 0.002563 | 0.056368 | -1.3116 | down |
| hsa-miR-199b-5p | 5.70E-07 | 6.52E-05 | -1.30881 | down |
| hsa-miR-4443 | 0.010144 | 0.146833 | -1.30376 | down |
| hsa-miR-5100 | 0.001128 | 0.032256 | -1.29678 | down |
| hsa-miR-590-3p | 8.72E-07 | 9.50E-05 | -1.27301 | down |
| novel_miR_835 | 0.013153 | 0.177393 | -1.25549 | down |
| hsa-miR-624-5p | 1.27E-06 | 0.000121 | -1.25432 | down |
| hsa-miR-136-3p | 0.013802 | 0.183518 | -1.24216 | down |
| novel_miR_666 | 0.011099 | 0.156684 | -1.20316 | down |
| hsa-miR-146a-3p | 0.009704 | 0.14226 | -1.17388 | down |
| novel_miR_777 | 0.010759 | 0.154756 | -1.13765 | down |
| novel_miR_765 | 0.026824 | 0.278613 | -1.10417 | down |
| hsa-miR-31-5p | 0.018486 | 0.228528 | -1.09044 | down |
| novel_miR_828 | 0.008877 | 0.133563 | -1.08764 | down |
| hsa-miR-3684 | 0.025614 | 0.272265 | -1.08365 | down |
| hsa-miR-598-3p | 4.37E-05 | 0.002377 | -1.0819 | down |
| hsa-miR-369-5p | 0.033586 | 0.321381 | -1.07983 | down |
| novel_miR_604 | 4.76E-05 | 0.002531 | -1.06824 | down |
| hsa-miR-106a-3p | 0.000401 | 0.014324 | -1.06699 | down |
| hsa-miR-374a-5p | 1.70E-09 | 4.86E-07 | -1.05733 | down |
| novel_miR_519 | 6.05E-05 | 0.002946 | -1.03694 | down |
| hsa-miR-125b-5p | 0.004972 | 0.0901 | -1.03271 | down |
| novel_miR_425 | 0.039829 | 0.356866 | -1.00887 | down |
| novel_miR_298 | 0.000122 | 0.005384 | -1.00792 | down |
| novel_miR_556 | 0.000122 | 0.005384 | -1.00792 | down |
| hsa-miR-335-3p | 5.02E-05 | 0.002552 | -1.00478 | down |
| novel_miR_227 | 0.035531 | 0.337177 | -1.00391 | down |
| novel_miR_49 | 0.039277 | 0.355046 | -0.98512 | down |
| hsa-miR-514a-3p | 0.005817 | 0.098549 | -0.97963 | down |
| hsa-miR-4433b-3p | 0.00369 | 0.071396 | -0.97934 | down |
| hsa-miR-126-5p | 6.00E-06 | 0.00049 | -0.97405 | down |
| hsa-miR-20a-5p | 1.48E-08 | 3.08E-06 | -0.96947 | down |
| hsa-miR-499a-5p | 0.000125 | 0.005407 | -0.96704 | down |
| hsa-miR-340-5p | 7.26E-09 | 1.66E-06 | -0.95999 | down |
| hsa-miR-301a-5p | 8.33E-05 | 0.003888 | -0.95584 | down |
| hsa-miR-29a-3p | 1.96E-07 | 2.80E-05 | -0.95554 | down |
| novel_miR_668 | 0.048382 | 0.37383 | -0.94145 | down |
| hsa-miR-335-5p | 1.76E-05 | 0.001218 | -0.93725 | down |
| hsa-miR-873-5p | 0.046151 | 0.363212 | -0.93596 | down |
| novel_miR_233 | 0.048848 | 0.376145 | -0.9264 | down |
| hsa-miR-486-3p | 0.025517 | 0.272265 | -0.92519 | down |
| hsa-miR-548n | 0.037386 | 0.346163 | -0.90794 | down |
| hsa-miR-548aq-3p | 0.029554 | 0.291611 | -0.90568 | down |
| novel_miR_141 | 0.029582 | 0.291611 | -0.90253 | down |
| novel_miR_446 | 0.0404 | 0.356866 | -0.9002 | down |
| novel_miR_532 | 0.000419 | 0.014739 | -0.89064 | down |
| novel_miR_400 | 0.042823 | 0.356866 | -0.89046 | down |
| hsa-miR-181a-3p | 0.003842 | 0.073217 | -0.87634 | down |
| hsa-miR-340-3p | 4.33E-07 | 5.22E-05 | -0.87595 | down |
| novel_miR_47 | 0.037343 | 0.346163 | -0.87141 | down |
| hsa-miR-32-5p | 3.64E-05 | 0.002135 | -0.8704 | down |
| hsa-miR-628-5p | 0.006905 | 0.111641 | -0.86785 | down |
| hsa-miR-3909 | 0.003054 | 0.06528 | -0.86777 | down |
| novel_miR_219 | 0.041071 | 0.356866 | -0.86426 | down |
| hsa-miR-450b-5p | 0.003642 | 0.071396 | -0.85906 | down |
| hsa-miR-5695 | 0.00073 | 0.022865 | -0.8588 | down |
| hsa-miR-301a-3p | 0.004773 | 0.087916 | -0.85283 | down |
| hsa-miR-660-5p | 3.97E-05 | 0.002214 | -0.85124 | down |
| hsa-miR-141-3p | 0.041446 | 0.356866 | -0.82626 | down |
| hsa-miR-4677-5p | 0.02493 | 0.270641 | -0.82335 | down |
| hsa-miR-548al | 0.021279 | 0.242112 | -0.81174 | down |
| hsa-miR-24-1-5p | 0.005468 | 0.094958 | -0.80851 | down |
| hsa-miR-125a-5p | 0.003522 | 0.071396 | -0.80351 | down |
| hsa-miR-450a-5p | 0.003574 | 0.071396 | -0.79547 | down |
| hsa-miR-2277-5p | 0.040811 | 0.356866 | -0.78112 | down |
| hsa-miR-99a-5p | 0.000448 | 0.015293 | -0.77198 | down |
| hsa-miR-126-3p | 9.84E-06 | 0.000726 | -0.77051 | down |
| hsa-miR-1260b | 0.009801 | 0.142766 | -0.76117 | down |
| hsa-miR-18b-5p | 0.005481 | 0.094958 | -0.7561 | down |
| hsa-miR-4742-5p | 0.045246 | 0.360333 | -0.73976 | down |
| hsa-miR-142-3p | 0.000267 | 0.010166 | -0.73069 | down |
| hsa-let-7f-5p | 2.75E-06 | 0.000252 | -0.72654 | down |
| hsa-miR-188-5p | 0.044443 | 0.356866 | -0.72162 | down |
| hsa-miR-618 | 0.017742 | 0.222939 | -0.72142 | down |
| hsa-miR-21-5p | 8.38E-06 | 0.000661 | -0.7201 | down |
| hsa-miR-339-5p | 0.002105 | 0.049624 | -0.71004 | down |
| hsa-miR-3143 | 0.008459 | 0.129835 | -0.7076 | down |
| hsa-miR-190a-5p | 0.002533 | 0.05624 | -0.70521 | down |
| hsa-miR-29b-3p | 4.97E-05 | 0.002552 | -0.70402 | down |
| hsa-miR-181a-2-3p | 3.58E-05 | 0.002135 | -0.70181 | down |
| hsa-miR-1278 | 0.000108 | 0.004959 | -0.69762 | down |
| hsa-miR-18a-5p | 0.000177 | 0.007098 | -0.6942 | down |
| novel_miR_836 | 0.000294 | 0.011038 | -0.69007 | down |
| novel_miR_819 | 0.001225 | 0.034584 | -0.67108 | down |
| hsa-miR-451a | 0.001848 | 0.045437 | -0.66996 | down |
| novel_miR_700 | 0.00134 | 0.036934 | -0.66656 | down |
| hsa-miR-9903 | 0.023859 | 0.263605 | -0.66279 | down |
| hsa-miR-27b-3p | 1.08E-05 | 0.000771 | -0.65737 | down |
| hsa-miR-424-5p | 0.044436 | 0.356866 | -0.65274 | down |
| hsa-miR-144-3p | 0.002151 | 0.050204 | -0.65078 | down |
| hsa-miR-5001-3p | 0.04047 | 0.356866 | -0.64005 | down |
| hsa-miR-98-5p | 7.46E-08 | 1.14E-05 | -0.63998 | down |
| hsa-miR-32-3p | 0.030264 | 0.294964 | -0.63827 | down |
| hsa-miR-374a-3p | 0.005091 | 0.090257 | -0.63032 | down |
| hsa-miR-24-2-5p | 0.000565 | 0.018464 | -0.62941 | down |
| hsa-miR-101-3p | 0.000341 | 0.012375 | -0.6289 | down |
| hsa-miR-651-5p | 0.001594 | 0.041418 | -0.62681 | down |
| hsa-miR-152-3p | 0.038122 | 0.351549 | -0.62653 | down |
| hsa-miR-28-5p | 0.002457 | 0.055635 | -0.62095 | down |
| hsa-miR-581 | 0.003249 | 0.067548 | -0.6091 | down |
| hsa-miR-1271-5p | 0.047135 | 0.368898 | -0.60317 | down |
| hsa-miR-5193 | 0.000129 | 0.005457 | 1.929473 | up |
| hsa-miR-5003-3p | 0.002596 | 0.056533 | 1.49168 | up |
| hsa-miR-3127-5p | 0.003701 | 0.071396 | 1.472351 | up |
| hsa-miR-5690 | 0.00098 | 0.028745 | 1.470262 | up |
| novel_miR_96 | 0.003655 | 0.071396 | 1.445824 | up |
| hsa-miR-6734-5p | 1.89E-05 | 0.001272 | 1.444353 | up |
| hsa-miR-122-5p | 0.004604 | 0.086312 | 1.427898 | up |
| hsa-miR-4755-3p | 0.005003 | 0.0901 | 1.364167 | up |
| hsa-miR-320a-3p | 3.62E-09 | 9.20E-07 | 1.346963 | up |
| novel_miR_562 | 0.004766 | 0.087916 | 1.340167 | up |
| hsa-miR-5009-5p | 3.31E-05 | 0.002045 | 1.338308 | up |
| novel_miR_89 | 0.006312 | 0.105363 | 1.296268 | up |
| novel_miR_274 | 0.009054 | 0.135335 | 1.276085 | up |
| novel_miR_358 | 0.003207 | 0.067548 | 1.222355 | up |
| hsa-miR-939-5p | 9.35E-07 | 9.72E-05 | 1.221384 | up |
| hsa-miR-4498 | 0.016339 | 0.208765 | 1.214836 | up |
| hsa-miR-3177-3p | 0.000514 | 0.017187 | 1.184024 | up |
| hsa-miR-6509-3p | 0.019591 | 0.231627 | 1.178338 | up |
| novel_miR_336 | 0.019648 | 0.231627 | 1.166489 | up |
| novel_miR_65 | 0.019648 | 0.231627 | 1.166489 | up |
| hsa-miR-6726-3p | 0.009169 | 0.136171 | 1.155045 | up |
| novel_miR_522 | 0.02118 | 0.242112 | 1.14844 | up |
| hsa-miR-2110 | 4.46E-10 | 1.46E-07 | 1.146324 | up |
| novel_miR_476 | 0.025821 | 0.272265 | 1.132844 | up |
| novel_miR_722 | 0.024344 | 0.267668 | 1.12257 | up |
| novel_miR_793 | 0.019918 | 0.232921 | 1.111905 | up |
| novel_miR_59 | 0.005715 | 0.097543 | 1.111462 | up |
| novel_miR_200 | 0.019458 | 0.231627 | 1.096043 | up |
| hsa-miR-6803-3p | 0.01187 | 0.165525 | 1.095318 | up |
| hsa-miR-320b | 3.78E-07 | 4.85E-05 | 1.095155 | up |
| hsa-miR-4685-3p | 0.000436 | 0.015102 | 1.091228 | up |
| novel_miR_315 | 0.002419 | 0.055315 | 1.087919 | up |
| novel_miR_705 | 0.027289 | 0.278613 | 1.081181 | up |
| hsa-miR-331-3p | 2.22E-08 | 3.90E-06 | 1.072456 | up |
| hsa-miR-6894-3p | 0.030309 | 0.294964 | 1.061704 | up |
| novel_miR_204 | 0.025088 | 0.270641 | 1.061246 | up |
| hsa-miR-3146 | 0.035978 | 0.338493 | 1.044722 | up |
| novel_miR_352 | 0.025838 | 0.272265 | 1.039116 | up |
| novel_miR_670 | 0.027167 | 0.278613 | 1.037739 | up |
| hsa-miR-1270 | 0.008689 | 0.131606 | 1.037732 | up |
| novel_miR_28 | 0.029569 | 0.291611 | 1.032361 | up |
| hsa-miR-4440 | 0.042825 | 0.356866 | 1.02972 | up |
| hsa-miR-1229-3p | 0.011958 | 0.165747 | 1.019551 | up |
| hsa-miR-5090 | 0.023337 | 0.259085 | 1.019227 | up |
| novel_miR_368 | 0.04334 | 0.356866 | 1.015481 | up |
| hsa-miR-5187-5p | 0.044472 | 0.356866 | 1.01536 | up |
| novel_miR_691 | 0.043779 | 0.356866 | 1.012544 | up |
| novel_miR_471 | 0.031778 | 0.306654 | 0.997691 | up |
| hsa-miR-3140-3p | 0.001465 | 0.038952 | 0.987205 | up |
| hsa-miR-299-3p | 0.044229 | 0.356866 | 0.979138 | up |
| hsa-miR-629-5p | 1.01E-06 | 0.000101 | 0.967243 | up |
| hsa-miR-3180 | 0.036262 | 0.338493 | 0.962199 | up |
| hsa-miR-3180-3p | 0.036262 | 0.338493 | 0.962199 | up |
| novel_miR_878 | 0.025953 | 0.272265 | 0.954244 | up |
| hsa-let-7b-3p | 0.001762 | 0.044778 | 0.953883 | up |
| novel_miR_795 | 0.038933 | 0.355046 | 0.953516 | up |
| novel_miR_594 | 0.013928 | 0.184129 | 0.951015 | up |
| hsa-miR-3127-3p | 0.012822 | 0.175586 | 0.938933 | up |
| novel_miR_137 | 0.002785 | 0.060077 | 0.933389 | up |
| novel_miR_702 | 0.040817 | 0.356866 | 0.932507 | up |
| hsa-miR-326 | 0.000756 | 0.023352 | 0.930544 | up |
| hsa-miR-15b-3p | 3.82E-07 | 4.85E-05 | 0.926719 | up |
| hsa-miR-4669 | 0.0481 | 0.37383 | 0.926644 | up |
| novel_miR_381 | 0.000133 | 0.005542 | 0.916058 | up |
| hsa-miR-423-5p | 2.10E-08 | 3.90E-06 | 0.915971 | up |
| novel_miR_500 | 0.044291 | 0.356866 | 0.914709 | up |
| novel_miR_180 | 0.041637 | 0.356866 | 0.914239 | up |
| hsa-miR-5010-5p | 0.018045 | 0.224289 | 0.908556 | up |
| hsa-miR-4664-3p | 0.043352 | 0.356866 | 0.905863 | up |
| hsa-miR-1343-3p | 0.027026 | 0.278613 | 0.899442 | up |
| hsa-miR-6855-5p | 0.029052 | 0.291409 | 0.898651 | up |
| hsa-miR-6842-5p | 0.043987 | 0.356866 | 0.889715 | up |
| hsa-miR-1224-5p | 0.006793 | 0.11097 | 0.887851 | up |
| hsa-miR-6799-3p | 0.025016 | 0.270641 | 0.885677 | up |
| hsa-miR-10401-3p | 0.017191 | 0.217809 | 0.871011 | up |
| novel_miR_287 | 0.040252 | 0.356866 | 0.861854 | up |
| hsa-miR-550a-3p | 0.002062 | 0.049122 | 0.861185 | up |
| hsa-miR-3615 | 8.16E-05 | 0.003886 | 0.845548 | up |
| hsa-miR-574-5p | 0.000306 | 0.011282 | 0.834058 | up |
| hsa-miR-877-5p | 0.00102 | 0.029521 | 0.824842 | up |
| hsa-miR-548at-5p | 0.02902 | 0.291409 | 0.822441 | up |
| novel_miR_786 | 0.046046 | 0.363212 | 0.82014 | up |
| hsa-let-7b-5p | 5.64E-05 | 0.002804 | 0.819462 | up |
| hsa-miR-16-2-3p | 0.003715 | 0.071396 | 0.807764 | up |
| hsa-miR-766-3p | 0.006788 | 0.11097 | 0.803975 | up |
| hsa-miR-6741-5p | 0.041852 | 0.356866 | 0.803571 | up |
| hsa-miR-1292-5p | 2.98E-05 | 0.001893 | 0.799775 | up |
| novel_miR_717 | 0.021643 | 0.243825 | 0.797595 | up |
| hsa-miR-1307-3p | 0.00128 | 0.0357 | 0.793847 | up |
| hsa-miR-6877-5p | 0.014487 | 0.190412 | 0.774674 | up |
| hsa-miR-184 | 0.013116 | 0.177393 | 0.763449 | up |
| hsa-miR-147b-3p | 0.000584 | 0.018723 | 0.751145 | up |
| hsa-miR-664a-5p | 0.021439 | 0.242727 | 0.742848 | up |
| hsa-miR-491-5p | 0.026494 | 0.276674 | 0.729068 | up |
| novel_miR_884 | 0.010841 | 0.154954 | 0.724656 | up |
| novel_miR_616 | 0.007247 | 0.115104 | 0.707898 | up |
| hsa-miR-486-5p | 0.000267 | 0.010166 | 0.705002 | up |
| novel_miR_617 | 0.007633 | 0.120394 | 0.700199 | up |
| hsa-let-7d-3p | 0.003434 | 0.070756 | 0.691029 | up |
| hsa-miR-3940-3p | 0.002507 | 0.056219 | 0.690943 | up |
| hsa-miR-636 | 0.003538 | 0.071396 | 0.686386 | up |
| hsa-miR-1285-5p | 0.047262 | 0.368898 | 0.681106 | up |
| hsa-miR-185-3p | 0.000821 | 0.025028 | 0.671823 | up |
| hsa-miR-6815-5p | 0.039251 | 0.355046 | 0.668146 | up |
| hsa-miR-532-3p | 0.001759 | 0.044778 | 0.657572 | up |
| hsa-miR-1273c | 0.027131 | 0.278613 | 0.635898 | up |
| hsa-miR-744-5p | 0.000223 | 0.008796 | 0.63526 | up |
| novel_miR_361 | 0.030295 | 0.294964 | 0.624199 | up |
| hsa-miR-4732-5p | 0.002196 | 0.050736 | 0.599644 | up |
| hsa-miR-652-3p | 0.001997 | 0.048579 | 0.599176 | up |
| novel_miR_119 | 0.019648 | 0.231627 | 0.598483 | up |
| novel_miR_375 | 0.019648 | 0.231627 | 0.598483 | up |
| hsa-miR-4746-5p | 0.005447 | 0.094958 | 0.593083 | up |
| novel_miR_355 | 0.048384 | 0.37383 | 0.586605 | up |

1. Plows JF, Stanley JL, Baker PN, Reynolds CM, Vickers MH (2018) The Pathophysiology of Gestational Diabetes Mellitus. Int J Mol Sci 19:<https://doi.org/10.3390/ijms19113342>

2. American Diabetes A (2018) 2. Classification and Diagnosis of Diabetes: Standards of Medical Care in Diabetes-2018. Diabetes Care 41:S13-S27. <https://doi.org/10.2337/dc18-S002>

3. Camelo Castillo W, Boggess K, Sturmer T, Brookhart MA, Benjamin DK, Jr., Jonsson Funk M (2015) Association of Adverse Pregnancy Outcomes With Glyburide vs Insulin in Women With Gestational Diabetes. JAMA Pediatr 169:452-458. <https://doi.org/10.1001/jamapediatrics.2015.74>

4. Feig DS, Moses RG (2011) Metformin therapy during pregnancy: good for the goose and good for the gosling too? Diabetes Care 34:2329-2330. <https://doi.org/10.2337/dc11-1153>

5. Dincgez B, Ercan I, Sahin I, Erturk NK (2024) The risk of developing gestational diabetes mellitus in maternal subclinical hypothyroidism: a systematic review and meta-analysis. Arch Gynecol Obstet 309:765-774. <https://doi.org/10.1007/s00404-023-07137-y>

6. Yogev Y, Xenakis EM, Langer O (2004) The association between preeclampsia and the severity of gestational diabetes: the impact of glycemic control. Am J Obstet Gynecol 191:1655-1660. <https://doi.org/10.1016/j.ajog.2004.03.074>

7. Group HSCR, Metzger BE, Lowe LP, Dyer AR, Trimble ER, Chaovarindr U, Coustan DR, Hadden DR, McCance DR, Hod M, McIntyre HD, Oats JJ, Persson B, Rogers MS, Sacks DA (2008) Hyperglycemia and adverse pregnancy outcomes. N Engl J Med 358:1991-2002. <https://doi.org/10.1056/NEJMoa0707943>

8. Federation IDJIdf (2017) IDF diabetes atlas 8th edition. 905-911.

9. Johns EC, Denison FC, Norman JE, Reynolds RM (2018) Gestational Diabetes Mellitus: Mechanisms, Treatment, and Complications. Trends Endocrinol Metab 29:743-754. <https://doi.org/10.1016/j.tem.2018.09.004>

10. Lende M, Rijhsinghani A (2020) Gestational Diabetes: Overview with Emphasis on Medical Management. Int J Environ Res Public Health 17:<https://doi.org/10.3390/ijerph17249573>

11. Filardi T, Catanzaro G, Mardente S, Zicari A, Santangelo C, Lenzi A, Morano S, Ferretti E (2020) Non-Coding RNA: Role in Gestational Diabetes Pathophysiology and Complications. Int J Mol Sci 21:<https://doi.org/10.3390/ijms21114020>

12. Anfossi S, Babayan A, Pantel K, Calin GA (2018) Clinical utility of circulating non-coding RNAs - an update. Nat Rev Clin Oncol 15:541-563. <https://doi.org/10.1038/s41571-018-0035-x>

13. Chen B, Huang S (2018) Circular RNA: An emerging non-coding RNA as a regulator and biomarker in cancer. Cancer Lett 418:41-50. <https://doi.org/10.1016/j.canlet.2018.01.011>

14. Ha M, Kim VN (2014) Regulation of microRNA biogenesis. Nat Rev Mol Cell Biol 15:509-524. <https://doi.org/10.1038/nrm3838>

15. Kozomara A, Birgaoanu M, Griffiths-Jones S (2019) miRBase: from microRNA sequences to function. Nucleic Acids Res 47:D155-D162. <https://doi.org/10.1093/nar/gky1141>

16. Peng Y, Croce CM (2016) The role of MicroRNAs in human cancer. Signal Transduct Target Ther 1:15004. <https://doi.org/10.1038/sigtrans.2015.4>

17. Soifer HS, Rossi JJ, Saetrom P (2007) MicroRNAs in disease and potential therapeutic applications. Mol Ther 15:2070-2079. <https://doi.org/10.1038/sj.mt.6300311>

18. Guay C, Regazzi R (2016) New emerging tasks for microRNAs in the control of beta-cell activities. Biochim Biophys Acta 1861:2121-2129. <https://doi.org/10.1016/j.bbalip.2016.05.003>

19. Liu ZN, Jiang Y, Liu XQ, Yang MM, Chen C, Zhao BH, Huang HF, Luo Q (2021) MiRNAs in Gestational Diabetes Mellitus: Potential Mechanisms and Clinical Applications. J Diabetes Res 2021:4632745. <https://doi.org/10.1155/2021/4632745>

20. Chen DB, Wang W (2013) Human placental microRNAs and preeclampsia. Biol Reprod 88:130. <https://doi.org/10.1095/biolreprod.113.107805>

21. Iljas JD, Guanzon D, Elfeky O, Rice GE, Salomon C (2017) Review: Bio-compartmentalization of microRNAs in exosomes during gestational diabetes mellitus. Placenta 54:76-82. <https://doi.org/10.1016/j.placenta.2016.12.002>

22. Cao JL, Zhang L, Li J, Tian S, Lv XD, Wang XQ, Su X, Li Y, Hu Y, Ma X, Xia HF (2016) Up-regulation of miR-98 and unraveling regulatory mechanisms in gestational diabetes mellitus. Sci Rep 6:32268. <https://doi.org/10.1038/srep32268>

23. Nair S, Jayabalan N, Guanzon D, Palma C, Scholz-Romero K, Elfeky O, Zuniga F, Ormazabal V, Diaz E, Rice GE, Duncombe G, Jansson T, McIntyre HD, Lappas M, Salomon C (2018) Human placental exosomes in gestational diabetes mellitus carry a specific set of miRNAs associated with skeletal muscle insulin sensitivity. Clin Sci (Lond) 132:2451-2467. <https://doi.org/10.1042/CS20180487>

24. Nguyen-Ngo C, Jayabalan N, Salomon C, Lappas M (2019) Molecular pathways disrupted by gestational diabetes mellitus. J Mol Endocrinol 63:R51-R72. <https://doi.org/10.1530/JME-18-0274>

25. Ahn M, Yoder SM, Wang Z, Oh E, Ramalingam L, Tunduguru R, Thurmond DC (2016) The p21-activated kinase (PAK1) is involved in diet-induced beta cell mass expansion and survival in mice and human islets. Diabetologia 59:2145-2155. <https://doi.org/10.1007/s00125-016-4042-0>

26. Li L, Wang S, Li H, Wan J, Zhou Q, Zhou Y, Zhang C (2018) microRNA-96 protects pancreatic beta-cell function by targeting PAK1 in gestational diabetes mellitus. Biofactors 44:539-547. <https://doi.org/10.1002/biof.1461>

27. Livak KJ, Schmittgen TD (2001) Analysis of relative gene expression data using real-time quantitative PCR and the 2(-Delta Delta C(T)) Method. Methods 25:402-408. <https://doi.org/10.1006/meth.2001.1262>

28. Friedlander MR, Mackowiak SD, Li N, Chen W, Rajewsky N (2012) miRDeep2 accurately identifies known and hundreds of novel microRNA genes in seven animal clades. Nucleic Acids Res 40:37-52. <https://doi.org/10.1093/nar/gkr688>

29. Fernandez-Valverde SL, Taft RJ, Mattick JS (2011) MicroRNAs in beta-cell biology, insulin resistance, diabetes and its complications. Diabetes 60:1825-1831. <https://doi.org/10.2337/db11-0171>

30. Zhao C, Dong J, Jiang T, Shi Z, Yu B, Zhu Y, Chen D, Xu J, Huo R, Dai J, Xia Y, Pan S, Hu Z, Sha J (2011) Early second-trimester serum miRNA profiling predicts gestational diabetes mellitus. PLoS One 6:e23925. <https://doi.org/10.1371/journal.pone.0023925>

31. Wang P, Wang Z, Liu G, Jin C, Zhang Q, Man S, Wang Z (2019) miR-657 Promotes Macrophage Polarization toward M1 by Targeting FAM46C in Gestational Diabetes Mellitus. Mediators Inflamm 2019:4851214. <https://doi.org/10.1155/2019/4851214>

32. Zhang YL, Chen XQ (2020) Dysregulation of microRNA-770-5p influences pancreatic-beta-cell function by targeting TP53 regulated inhibitor of apoptosis 1 in gestational diabetes mellitus. Eur Rev Med Pharmacol Sci 24:793-801. <https://doi.org/10.26355/eurrev_202001_20062>

33. Zhou X, Xiang C, Zheng X (2019) miR-132 serves as a diagnostic biomarker in gestational diabetes mellitus and its regulatory effect on trophoblast cell viability. Diagn Pathol 14:119. <https://doi.org/10.1186/s13000-019-0899-9>

34. Song Z, Guo Q, Wang H, Gao L, Wang S, Liu D, Liu J, Qi Y, Lin B (2020) miR-5193, regulated by FUT1, suppresses proliferation and migration of ovarian cancer cells by targeting TRIM11. Pathol Res Pract 216:153148. <https://doi.org/10.1016/j.prp.2020.153148>

35. Pan Y, Zhang R, Chen H, Chen W, Wu K, Lv J (2019) Expression of Tripartite Motif-Containing Proteactiin 11 (TRIM11) is Associated with the Progression of Human Prostate Cancer and is Downregulated by MicroRNA-5193. Med Sci Monit 25:98-106. <https://doi.org/10.12659/MSM.911818>

36. Yang M, Hu H, Wu S, Ding J, Yin B, Huang B, Li F, Guo X, Han L (2022) EIF4A3-regulated circ_0087429 can reverse EMT and inhibit the progression of cervical cancer via miR-5003-3p-dependent upregulation of OGN expression. J Exp Clin Cancer Res 41:165. <https://doi.org/10.1186/s13046-022-02368-4>

37. Li J, Han J, Zhao A, Zhang G (2022) CircPAPPA Regulates the Proliferation, Migration, Invasion, Apoptosis, and Cell Cycle of Trophoblast Cells Through the miR-3127-5p/HOXA7 Axis. Reprod Sci 29:1215-1225. <https://doi.org/10.1007/s43032-021-00802-0>

38. Wang R, Du X, Zhi Y (2020) Screening of Critical Genes Involved in Metastasis and Prognosis of High-Grade Serous Ovarian Cancer by Gene Expression Profile Data. J Comput Biol 27:1104-1114. <https://doi.org/10.1089/cmb.2019.0235>

39. Zhang D, Yang Y, Kang Y, Xie D, Zhang X, Hao J (2023) Dysregulated expression of microRNA involved in resistance to osimertinib in EGFR mutant non-small cell lung cancer cells. J Thorac Dis 15:1978-1993. <https://doi.org/10.21037/jtd-23-401>

40. Lee HM, Wong WKK, Fan B, Lau ES, Hou Y, O CK, Luk AOY, Chow EYK, Ma RCW, Chan JCN, Kong APS (2021) Detection of increased serum miR-122-5p and miR-455-3p levels before the clinical diagnosis of liver cancer in people with type 2 diabetes. Sci Rep 11:23756. <https://doi.org/10.1038/s41598-021-03222-x>

41. Gillet V, Ouellet A, Stepanov Y, Rodosthenous RS, Croft EK, Brennan K, Abdelouahab N, Baccarelli A, Takser L (2019) miRNA Profiles in Extracellular Vesicles From Serum Early in Pregnancies Complicated by Gestational Diabetes Mellitus. J Clin Endocrinol Metab 104:5157-5169. <https://doi.org/10.1210/jc.2018-02693>

42. Baldwin AS, Jr. (1996) The NF-kappa B and I kappa B proteins: new discoveries and insights. Annu Rev Immunol 14:649-683. <https://doi.org/10.1146/annurev.immunol.14.1.649>

43. Feng H, Su R, Song Y, Wang C, Lin L, Ma J, Yang H (2016) Positive Correlation between Enhanced Expression of TLR4/MyD88/NF-kappaB with Insulin Resistance in Placentae of Gestational Diabetes Mellitus. PLoS One 11:e0157185. <https://doi.org/10.1371/journal.pone.0157185>

44. Ma Q, Fan J, Wang J, Yang S, Cong Q, Wang R, Lv Q, Liu R, Ning G (2015) High levels of chorionic gonadotrophin attenuate insulin sensitivity and promote inflammation in adipocytes. J Mol Endocrinol 54:161-170. <https://doi.org/10.1530/JME-14-0284>

45. Vasselon T, Detmers PA (2002) Toll receptors: a central element in innate immune responses. Infect Immun 70:1033-1041. <https://doi.org/10.1128/IAI.70.3.1033-1041.2002>

46. Mrizak I, Grissa O, Henault B, Fekih M, Bouslema A, Boumaiza I, Zaouali M, Tabka Z, Khan NA (2014) Placental infiltration of inflammatory markers in gestational diabetic women. Gen Physiol Biophys 33:169-176. <https://doi.org/10.4149/gpb_2013075>

47. Sati L, Soygur B, Celik-Ozenci C (2016) Expression of Mammalian Target of Rapamycin and Downstream Targets in Normal and Gestational Diabetic Human Term Placenta. Reprod Sci 23:324-332. <https://doi.org/10.1177/1933719115602765>

48. Woodgett JR (1990) Molecular cloning and expression of glycogen synthase kinase-3/factor A. EMBO J 9:2431-2438. <https://doi.org/10.1002/j.1460-2075.1990.tb07419.x>

49. Lappas M (2014) GSK3beta is increased in adipose tissue and skeletal muscle from women with gestational diabetes where it regulates the inflammatory response. PLoS One 9:e115854. <https://doi.org/10.1371/journal.pone.0115854>

50. Liong S, Lappas M (2015) Activation of AMPK improves inflammation and insulin resistance in adipose tissue and skeletal muscle from pregnant women. J Physiol Biochem 71:703-717. <https://doi.org/10.1007/s13105-015-0435-7>

51. Boyle KE, Hwang H, Janssen RC, DeVente JM, Barbour LA, Hernandez TL, Mandarino LJ, Lappas M, Friedman JE (2014) Gestational diabetes is characterized by reduced mitochondrial protein expression and altered calcium signaling proteins in skeletal muscle. PLoS One 9:e106872. <https://doi.org/10.1371/journal.pone.0106872>

52. Lappas M (2014) Activation of inflammasomes in adipose tissue of women with gestational diabetes. Mol Cell Endocrinol 382:74-83. <https://doi.org/10.1016/j.mce.2013.09.011>

53. Sirico A, Rossi ED, Degennaro VA, Arena V, Rizzi A, Tartaglione L, Di Leo M, Pitocco D, Lanzone A (2023) Placental diabesity: placental VEGF and CD31 expression according to pregestational BMI and gestational weight gain in women with gestational diabetes. Arch Gynecol Obstet 307:1823-1831. <https://doi.org/10.1007/s00404-022-06673-3>

54. Whitley RJ, Kimberlin DW (2005) Herpes simplex encephalitis: children and adolescents. Semin Pediatr Infect Dis 16:17-23. <https://doi.org/10.1053/j.spid.2004.09.007>

55. Chiu YH, Macmillan JB, Chen ZJ (2009) RNA polymerase III detects cytosolic DNA and induces type I interferons through the RIG-I pathway. Cell 138:576-591. <https://doi.org/10.1016/j.cell.2009.06.015>

56. Lappas M (2015) Double stranded viral RNA induces inflammation and insulin resistance in skeletal muscle from pregnant women in vitro. Metabolism 64:642-653. <https://doi.org/10.1016/j.metabol.2015.02.002>

57. Kirwan JP, Hauguel-De Mouzon S, Lepercq J, Challier JC, Huston-Presley L, Friedman JE, Kalhan SC, Catalano PM (2002) TNF-alpha is a predictor of insulin resistance in human pregnancy. Diabetes 51:2207-2213. <https://doi.org/10.2337/diabetes.51.7.2207>
